# Hyaluronic Acid Induces Activation of the κ-Opioid Receptor

**DOI:** 10.1371/journal.pone.0055510

**Published:** 2013-01-28

**Authors:** Barbara Zavan, Letizia Ferroni, Carlotta Giorgi, Girolamo Calò, Paola Brun, Roberta Cortivo, Giovanni Abatangelo, Paolo Pinton

**Affiliations:** 1 Department of Biomedical Sciences, University of Padova, Padova, Italy; 2 Department of Morphology, Surgery and Experimental Medicine, Section of General Pathology, Interdisciplinary Center for the Study of Inflammation (ICSI) and LTTA Center, University of Ferrara, Ferrara, Italy; 3 Department Experimental and Clinical Medicine, Section of Pharmacology and Neuroscience Center, University of Ferrara, Ferrara, Italy; University of Arizona, United States of America

## Abstract

**Introduction:**

Nociceptive pain is one of the most common types of pain that originates from an injury involving nociceptors. Approximately 60% of the knee joint innervations are classified as nociceptive. The specific biological mechanism underlying the regulation of nociceptors is relevant for the treatment of symptoms affecting the knee joint. Intra-articular administration of exogenous hyaluronic acid (HA) in patients with osteoarthritis (OA) appears to be particularly effective in reducing pain and improving patient function.

**Methods:**

We performed an *in vitro* study conducted in CHO cells that expressed a panel of opioid receptors and in primary rat dorsal root ganglion (DRG) neurons to determine if HA induces the activation of opioid peptide receptors (OPr) using both aequorin and the fluorescent dye Fura-2/AM.

**Results:**

Selective agonists and antagonists for each OPr expressed on CHO cells were used to test the efficacy of our *in vitro* model followed by stimulation with HA. The results showed that HA induces stimulatory effects on the κ receptor (KOP). These effects of HA were also confirmed in rat DRG neurons, which express endogenously the OPr.

**Conclusions:**

HA activates the KOP receptor in a concentration dependent manner, with a pEC_50_ value of 7.57.

## Introduction

The word “pain” comes from the Latin “poena”, which mean a fine or a penalty. Pain is indeed an unpleasant stimulus that warns of an injury that either is immediately impending or has already occurred, such as touching a hot object. Pain experts have divided the physical causes of pain into two types: neuropathic and nociceptive pain. Whereas neuropathic pain refers to the dysfunction of either the peripheral (peripheral neuropathy) or central (central neuropathy) nervous systems, nociceptive pain (the more common of the two) refers to the discomfort that results when a stimulus causes tissue damage to the muscles, bones, skin or internal organs [1]. An endless variety of stimuli can trigger nociceptive pain by activating nociceptors found primarily in the skin, joints (somatic sources) or the walls of organs (visceral sources). Nociceptors detect any type of stimuli and are localized on nerve endings throughout the body outside of the spinal cord. Though nociceptors are scarce in organs deep within the body, they are highly concentrated on the skin and joints [2].

The most common form of joint damage is OA, which is accompanied by significant joint pain [3]. As approximately 60% of knee joint innervation is classified as nociceptive, the specific biological mechanism underlying the regulation of nociceptors are relevant to the symptomatic treatment of this disease [4].

The intra-articular injection of morphine and opioid compounds appears to be somewhat effective in the treatment of pain in various arthritides without producing major side effects. Studies by several independent groups indicated that the application of morphine and opioid compounds might function through their receptors (OPr) in the periphery [5]–[8]. Three types of classic OPr have been identified: κ (KOP), δ (DOP), and µ (MOP). They are members of the G protein-coupled receptors (GPCRs) superfamily and may be further classified into several subgroups based on their respective ligand binding affinity [9], [10]. Evidence for the presence of OPr in the synovial tissue came first from pharmacological binding studies. It was found that human synovial tissues possess binding sites for the selective MOP antagonist naloxone [5], endorphin, met-enkephalin [6], and morphine [11]. More recently, MOP was identified in the synovial lining and sublining cells of patients with rheumatoid arthritis.

Nociceptive sensitivity is modulated by a large variety of mediators in the extracellular space. These mediators activate a large number of receptor classes, which in turn activate a plethora of signaling cascades [12]. How this multitude of cascades mediates nociceptor sensitization and pain is still poorly understood.

Recent studies have discovered the importance of the extracellular matrix (ECM) components in the modulation of nociception [13]. Intra-articular administration of exogenous HA appears to be effective in reducing pain and improving function in patients with OA. Moreover, this type of viscosupplementation (VS) was approved by the Food and Drug Administration in 1997 [14]. The analgesic and rewarding properties of opioid drugs occur through the activation of OPr. In addition to their central actions, opioid peptides/drugs may activate peripheral OPr and produce analgesic effects by inhibiting the excitability of sensory nerves and/or the release of proinflammatory neuropeptides [15].

In light of these clinical considerations, we have investigated if HA induces the activation of opioid peptide receptors (OPr).

To study this effect, CHO cell lines stably expressing the classical opioid receptor types DOP, KOP, and MOP as well as the nociceptin/orphanin FQ (N/OFQ) peptide (NOP) receptor, were tested. OPr are Gi-coupled receptors that alter the concentration of cyclic AMP (cAMP) and, like many other G protein-coupled receptors, can undergo rapid desensitization and internalization following exposure to agonist [16].

GPCRs that are coupled to cAMP require complex and less efficient screening methods such ligand binding assays or radioimmunoassay to measure activity compared to the GPCRs linked to phospholipase C activation and calcium (Ca^2^) release from intracellular stores. To overcome this technical complication, we decided to couple the activation of OPr to Ca^2+^ signals using CHO cell lines expressing the promiscuous G protein Gα_qi5_, which can couple any GPCR to an increase in cytosolic Ca^2+^ concentration ([Ca^2+^]_c_) [17]. This strategy to study OPr activation has been well documented [18].

The OPr are expressed in the peripheral terminals of the nociceptors [19]. Therefore, we confirmed our results in DRG neurons to address the criticism that all of the experiments were performed using only a heterologous expression system.

## Materials and Methods

### Drugs and reagents

Hyaluronic acid used in this study was from FIDIA Italy. Cells were stimulated with HA of two Molecular Weigh (MW): small MW: 6 kDa; medium MW: 200 kDa (as ponderal average molecular weight) at increasing concentrations comprised between 0,005 and 5 mg/ml. As selective agonists the following molecules were used: N/OFQ for the NOP line; [d-Pen2,d-Pen5]-Enkephalin (DPDPE) for the MOP line, dermorphin for the DOP line, and dynorphin A for the KOP line. norbinaltorphimine (norBNI) has been used as KOP receptor antagonist. As negative control it was used the buffer (KRB) without agonist.

Tissue culture media and supplements were from Invitrogen (Carlsbad, CA, USA) and from Cambrex Bioscience (Walkersville, Maryland, USA). All other reagents were from Sigma Chemical (Poole, U.K.).

### Cell cultures and transfection

CHO stable clones expressing DOP, KOP, MOP, NOP receptors and stably expressing the Gα_qi5_ protein were generated as previously described [20] and were cultured in DMEM and Ham F-12 (1∶1) supplemented with 5% fetal calf serum, penicillin (100 IU/ml), streptomycin (100 µg/ml) and fungizone (2.5 µg/ml). Stock cultures were further supplemented with geneticin (G418, 200 µg/ml) and hygromycin B (200 µg/ml).

For transfection, CHO cells were seeded 48 h prior to transfection onto different sized glass coverslips depending on the assay: 13 mm diameter for the aequorin experiments and 24 mm diameter for the Fura-2/AM measurements. Cells were allowed to grow to 50% confluence followed by transfection with a standard Ca^2+^-phosphate procedure. All experiments were performed 36 h post-transfection.

Isolation, dissociation plating and transfection of rat DRG neurons from adult rats (12 weeks) were performed as previously described [21], [22].

All cells were cultured at 37°C in humidified air containing 5% carbon dioxide.

### Ca^2+^ measurements

#### Aequorin measurement

Mitochondrial Ca^2+^ concentrations measurements were carried out as previously described [23]. Briefly, the cells were seeded onto 13-mm glass coverslips and allowed to grow to 75% confluence. At this stage, transfection with 4 μg of mitochondrial targeted aequorin was carried out. 36 h after transfection in order to obtain the active form of aequorin, cells were incubated for 2 h at 37°C, in KRB (Krebs–Ringer modified buffer: NaCl 125 mM, KCl 5 mM, Na_3_PO_4_ 1 mM, MgSO_4_ 1 mM, glucose 5.5 mM, HEPES 20 mM, pH 7.4) supplemented with 5 μM coelenterazine. Then all measurements were performed using an automatized luminometer (MicrobetaJET, PerkinElmer, CA, USA). KRB additioned of the different HA was then injected and luminescence was recorded for 60 s. To terminate the experiments and discharge the remaining aequorin for the normalization of the values obtained, a hypotonic solution containing 500 μM digitonin and 50 mM CaCl_2_ was injected. The results are expressed as % of probe discharged ± standard error (SE). This is possible because, when Ca^2+^ ions bind to three high-affinity sites (EF-hand type), aequorin undergoes an irreversible reaction, in which a photon is emitted and the resulting protein is inactive (discharged).

To convert the aequorin luminescence data into [Ca^2+^] values, computer algorithm based on the Ca^2+^ response curve of aequorins has been used as previously described [23].

#### Fura-2/AM measurements

Cytosolic free [Ca^2+^] were evaluated using the fluorescent Ca^2+^ indicator Fura-2/AM (Molecular Probes, Inc.). Briefly, cells were incubated in medium supplemented with 2.5 μM Fura-2/AM for 30 min, washed with KRB to remove extracellular probe and placed in a thermostatic incubation chamber at 37°C on the stage of an inverted fluorescence microscope (Zeiss Axiovert 200). Dynamic video imaging was performed using MetaFluor software (Universal Imaging Corp.). [Ca^2+^] calculated as previously described [24].

### Data analysis and terminology

The data were analyzed by nonlinear curve fitting equation using Graph Pad 4.0 software. The data are expressed as the mean ± SEM of at least 6 experiments and were analyzed statistically using one-way analysis of variance followed by Dunnett's test for multiple comparisons. Agonist potencies are given as pEC_50_ (the negative logarithm to base 10 of the molar concentration of an agonist that produces 50% of the maximal possible effect, E_max_). The antagonist potencies were derived from inhibition experiments and expressed as pK_B_ calculated from the following equation:
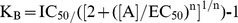
where IC_50_ is the concentration of antagonist that produces 50% inhibition of the agonist response, [A] is the concentration of agonist, EC_50_ is the concentration of agonist producing a 50% maximal response, and n is the Hill coefficient of the concentration response curve to the agonist [25].

## Results

To determine if HA is able to activate the OPr, we utilized CHO cells that expressed different opioid receptor types (e.g., NOP, DOP, MOP, KOP). To couple the activation of these receptors to Ca^2+^ signals instead of their native physiological second messenger cAMP, the cells were co-expressed with the promiscuous G protein Gα_qi5_, which can couple any GPCR to Ca^2+^ changes [17]. This results in the activation of the receptor inducing phospholipase C activation and the subsequent production of inositol 1,4,5 trisphosphate with Ca^2+^ release from the intracellular stores and an influx from the extracellular medium–this causes a transient rise in cytoplasmic [Ca^2+^] ([Ca^2+^]_c_) [26]. To demonstrate the validity of this approach, we measured [Ca^2+^]_c_ using the fluorescent cytosolic Ca^2+^ dye Fura-2/AM [24] as described in the Materials and Methods.

**Figure 1:**
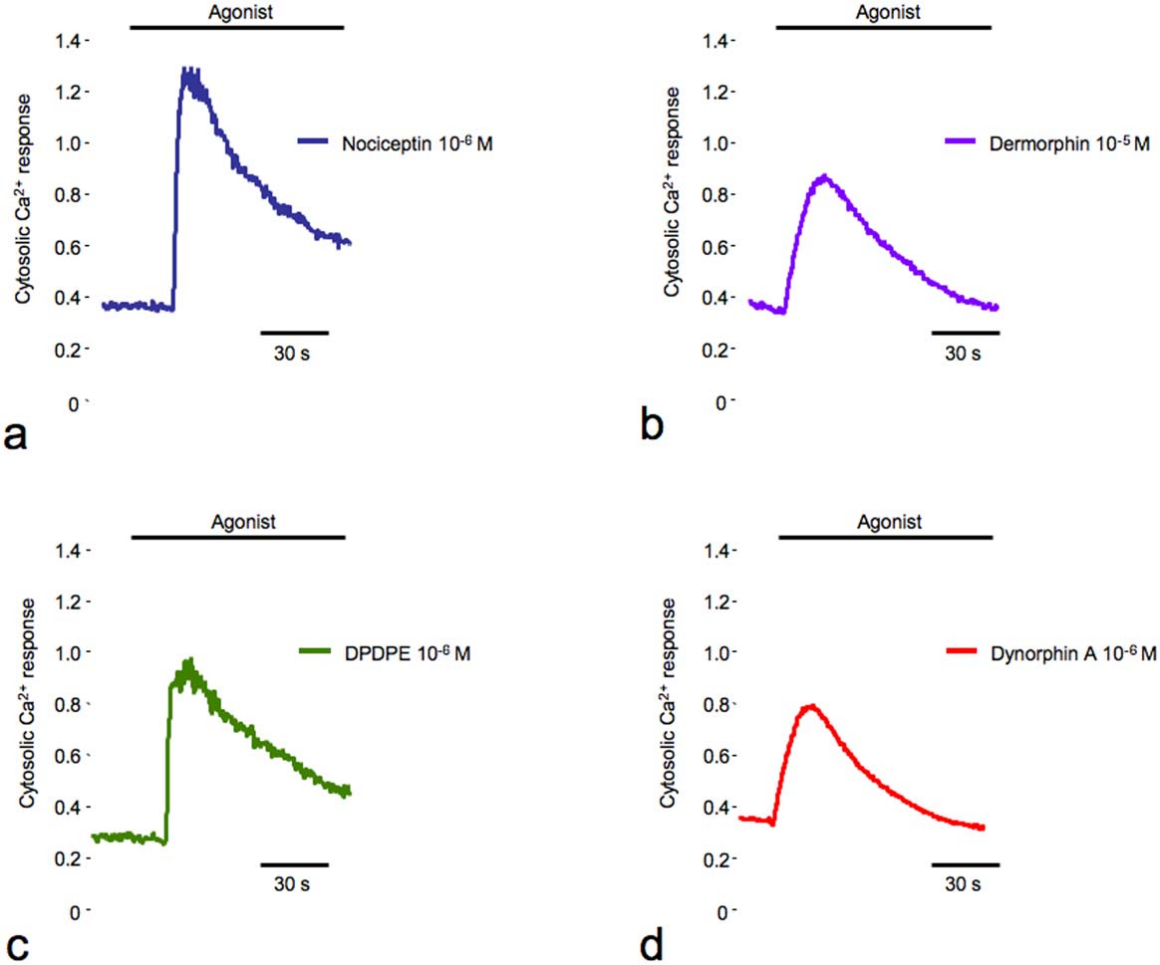
Cytosolic Ca^2+^ responses in CHO cell lines expressing human OPr. CHO stable clones expressing NOP, DOP, MOP, or KOP receptors were loaded with Fura-2/AM and stimulated with nociceptin (**a**), dermorphin (**b**), [d-Pen2,d-Pen5]-Enkephalin (DPDPE) (**c**) and dynorphin A (**d**) respectively, as indicated in figure. The kinetic behavior of the cytosolic Ca^2+^ response is presented as the 340 nm/380 nm ratio.

The following molecules were used as selective agonists: N/OFQ peptide (Nociceptin) for the NOP line; [d-Pen2,d-Pen5]-Enkephalin (DPDPE) for the DOP line, dermorphin for the MOP line and dynorphin A for the KOP line. All of the cell lines demonstrated a well-defined increase in [Ca^2+^]_c_ in the presence of their respective selective agonist (Fig. 1).

Though there are several methodologies to monitor changes in intracellular Ca^2+^ (e.g., fluorescent dyes, GFP-based indicators), the probe that offers the best signal/noise ratio is engineered aequorin, specifically aequorin expressed in the mitochondrial matrix. The aequorin signal increases proportionally almost to 3 log values of the Ca^2+^ concentration. For example, a rise in Ca^2+^ from 0,1 uM (typical of the either the mitochondrial matrix or the cytosol of a resting cell) to 1 µM (typical of the cytoplasm of an activated cell) or 10 µM (typical of the mitochondrial matrix upon activation) causes a 1000 and 100.000 fold increase, respectively, in aequorin luminescence. This is significantly larger compared to the best increase in signal achievable with fluorescent Ca^2+^ indicators (4–10-fold). Additionally, the increases in [Ca^2+^]_c_ cause a rapid increase in the [Ca^2+^] within the mitochondrial matrix ([Ca^2+^]_m_) where the probe is located, which usually exceeds the increase in the cytosol [23]. This approach is widely used for the identification of specific ligands of GPCRs because it is associated with a very high signal/noise ratio [27]–[30]. Based on these considerations, cells expressing mitochondria-targeted aequorin (mtAEQ) appear to provide a tool to screen for drugs that act on OPr.

**Figure 2:**
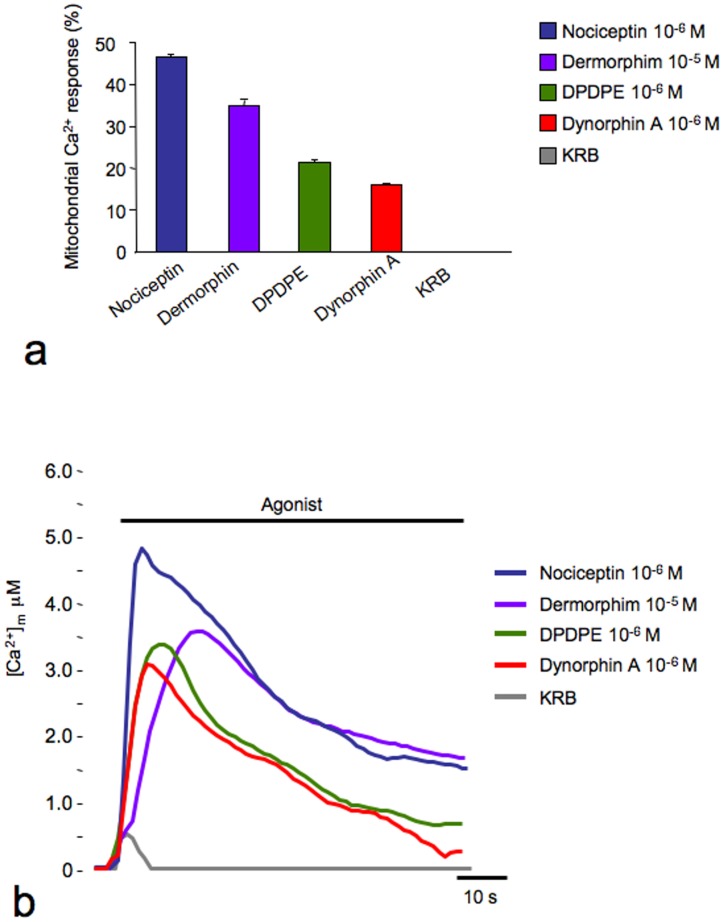
Mitochondrial Ca^2+^ responses in CHO cell lines expressing human OPr. CHO stable clones expressing NOP, DOP, MOP, or KOP receptors were transfected with a mitochondrial targeted aequorin probe and stimulated with nociceptin, [d-Pen2,d-Pen5]-Enkephalin (DPDPE), dermorphin and dynorphin A, respectively. The negative control was the addition of buffer (KRB) without agonist. The mitochondrial Ca^2+^ responses are expressed either as (**a**) percentage of probe discharged during the stimulation or (**b**) as [Ca^2+^] values.

As shown in Fig. 2, the selective agonists nociceptin, DPDPE, dermorphin and dynorphin A induce a specific and robust mitochondrial Ca^2+^ response upon binding with their respective receptors compared to the negative control (buffer KRB without agonist). The mitochondrial Ca^2+^ responses were expressed as either a percentage of probe discharged during the stimulation (Fig. 2a) or calibrated [Ca^2+^] values (Fig. 2b).

**Figure 3:**
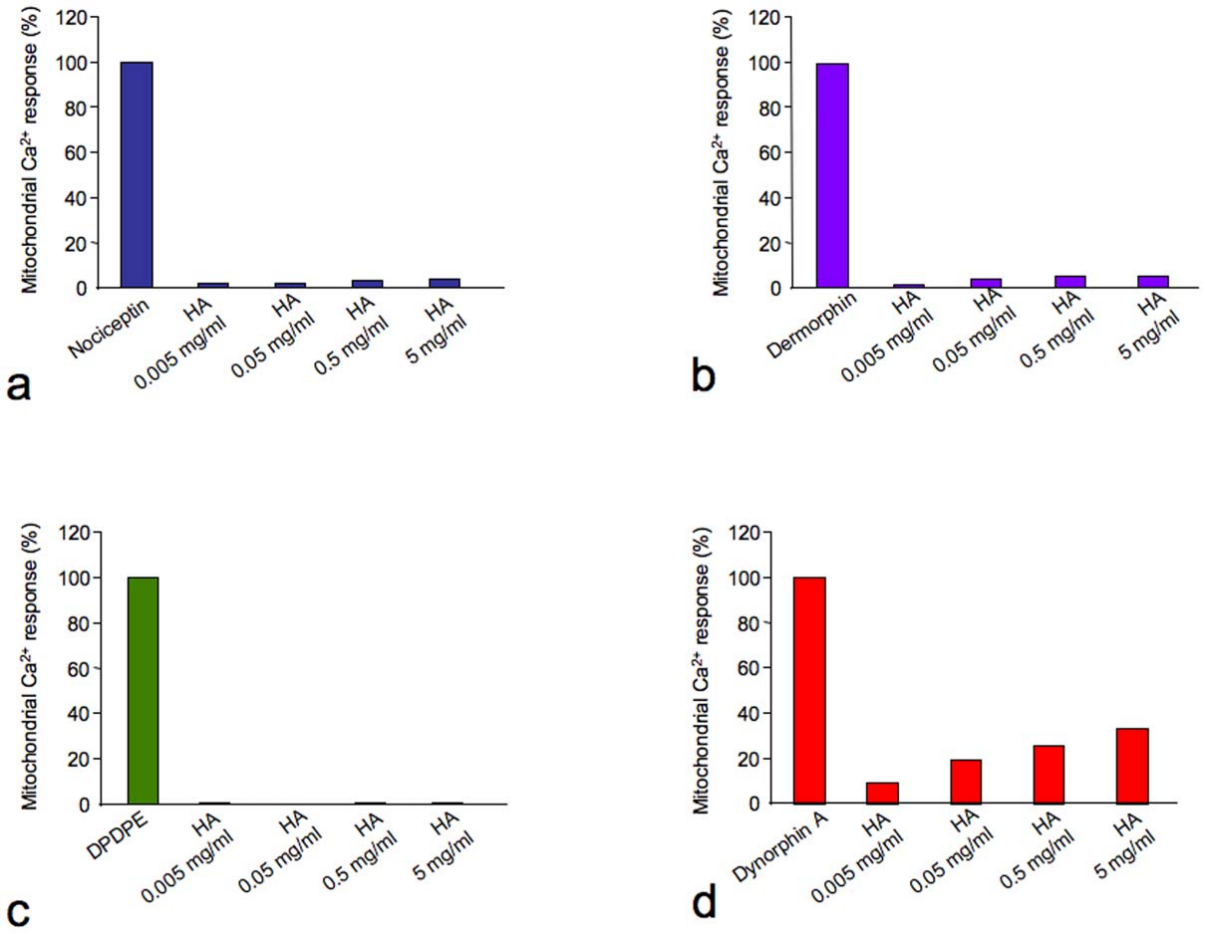
Analysis of the ability of HA to activate the NOP, DOP, MOP, **and** **KOP** **receptors.** CHO stable clones expressing NOP (**a**), DOP (**b**), MOP (**c**), or KOP (**d**) receptors were transfected with a mitochondrial targeted aequorin probe and stimulated with different HA concentrations as indicated in the figure. The mitochondrial Ca^2+^ responses are expressed as percentage of mitochondrial Ca^2+^ response compared to receptors stimulation with canonic agonist as indicated in the figure.

**Figure 4:**
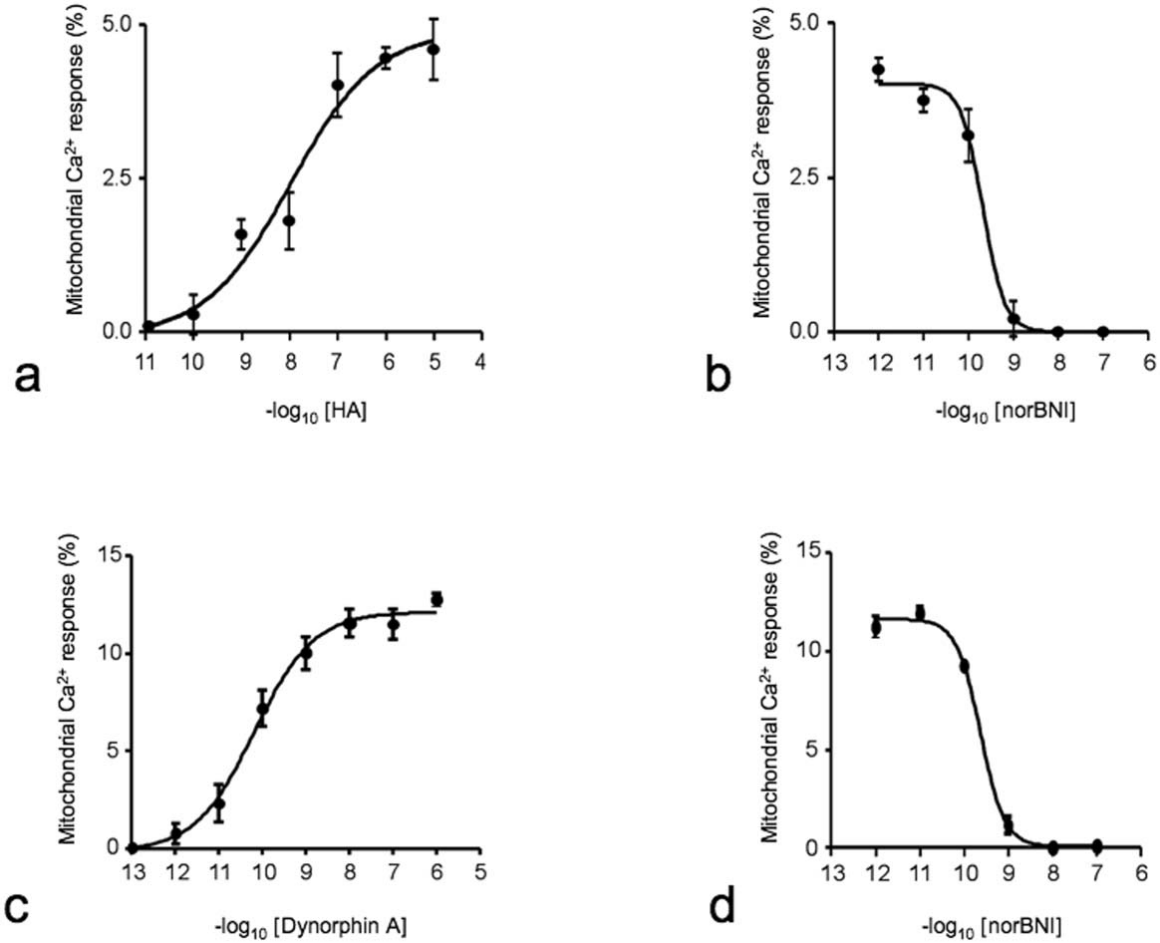
HA-dependent activation of the KOP receptor. (**a**) Dose response curve of HA on the CHO cells stably expressing KOP receptors. Cells were stimulated with 200 kDa HA at increasing concentrations ranging from 0,005 to 5 mg/ml. (**b**) Inhibition response curves of norbinaltor-phimine vs 200 kDa HA (5 mg/ml) in CHO cells stably expressing KOP receptors. (**c**) Dose response curve of dynorphin A on CHO cells stably expressing KOP receptors. (**d**) Inhibition response curves of norbinaltor-phimine vs dynorphin A (10^−9^ M) in CHO cells stably expressing KOP receptors. In all curves (**a–d**), the mitochondrial Ca^2+^ responses represent the percentage of probe discharged.

The same cell clones were stimulated with two types of HA (low molecular weight (MW) at 6 kDa and medium MW at 200 kDa) at concentrations ranging from 0,005 to 5 mg/ml. Though the smaller MW HA was unable to induce any significant light emission, the larger HA induced a significant detectable light emission only in KOP-expressing CHO cells (Fig. 3). This stimulatory effect was concentration dependent and had a pEC_50_ value of 7.57 (Fig. 4a and Fig. S1a). Fig. 4c (and Fig. S1c) reports the concentration-response curve to dynorphin A. As expected, the peptide induced a robust and concentration-dependent stimulatory effect showing high potency (pEC_50_ = 10.18). The maximal effects induced by HA were approximately half of those induced by dynorphin A; thus, HA appears to behave as a partial agonist at KOP receptors.

We then assessed the sensitivity of the KOP receptor antagonist norbinaltorphimine (norBNI) on the stimulatory effects of both dynorphin A and HA. As shown in Fig. 4b (and Fig. S1b), norBNI was able to inhibit the stimulatory effect of HA in a concentration-dependent manner, with a pKB value of 9.67. As shown in Fig. 4d (and Fig. S1d), similar results were observed with KOP clones treated with the antagonist in presence of dynorphin A (1 nM), with a calculated pKB value of 9.62.

**Figure 5:**
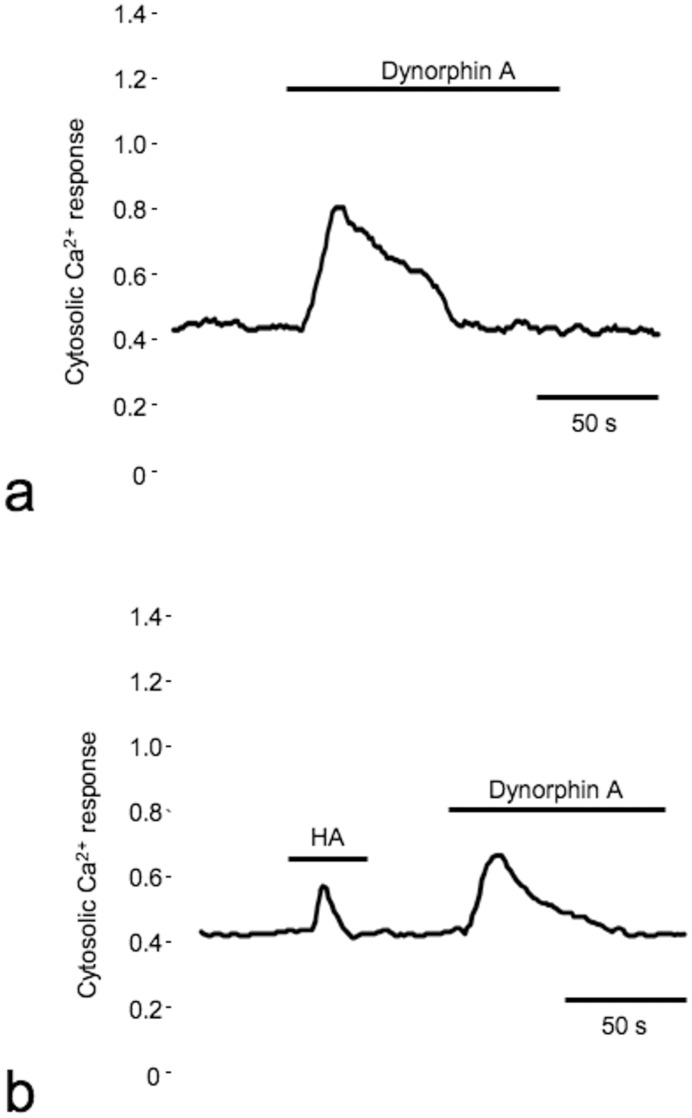
Cytosolic Ca^2+^ homeostasis in CHO cell lines expressing KOP receptors. CHO cells expressing KOP receptors were loaded with Fura-2/AM and stimulated with either dynorphin A [1 μM] alone (**a**) or with 200 kDa HA (5 mg/ml) and dynorphin A (**b**). The kinetic behavior of the cytosolic Ca^2+^ response is presented as the 340 nm/380 nm ratio.

To validate the results obtained from the mitochondrial aequorin-based assay, variations in the cytosolic [Ca^2+^]_c_ were investigated with the Fura-2/AM method in KOP cells. The stimulation of KOP-expressing CHO cells with the specific agonist dynorphin A (1 μM) resulted in an expected transient increase in [Ca^2+^]_c_ (Fig. 5a). As shown in Fig. 5b, a similar response was observed in cells stimulated with HA (200 kDa), though these responses are smaller in both amplitude (0.41±0.12 vs 0.15±0.09 ratio unit; n = 26) and duration (57±8 vs 11±14 s) respectively. The subsequent stimulation with dynorphin A induced a smaller transient increase in HA-pretreated cells compared to untreated cells. Analysis of the single traces indicated that the sum of area under the curve the cells treated with both HA and dynorphin A was comparable to that observed in cells stimulated only with dynorphin A. These data suggest that HA acts on the same signaling pathway as that of a canonical agonist, i.e., via selective activation of the KOP receptor.

We validated these observations in a more physiological cellular model by using rat DRG neurons that endogenously express different OPr, including KOP [31].

**Figure 6:**
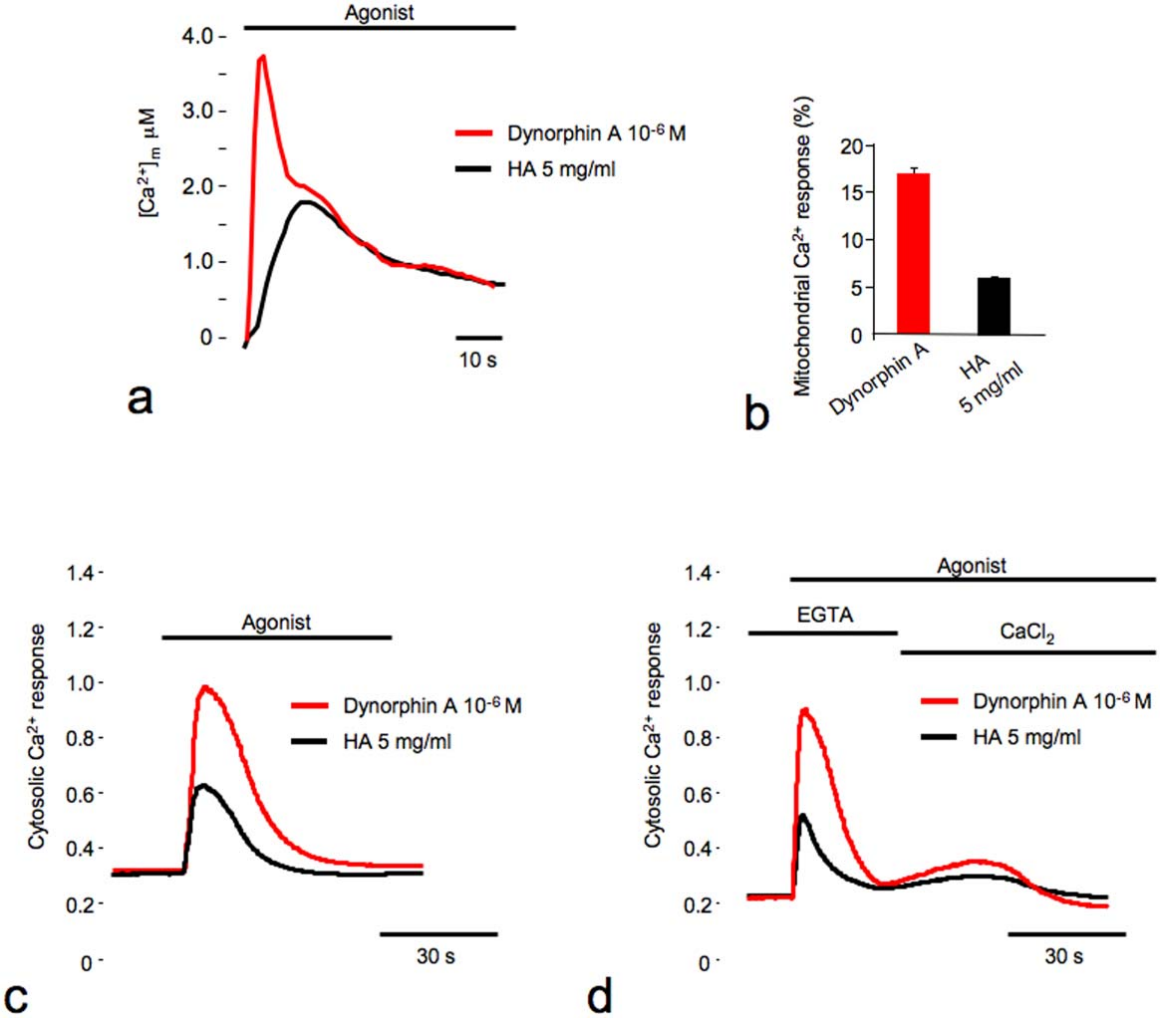
Analysis of cytosolic and mitochondrial Ca^2+^ responses on rat DRG neurons. (**a–b**) Rat DRG neurons were co-transfected with the mtAEQ probe and the promiscuous G protein Gα_qi5._ At 36 h post-transfection, the rat DRG neurons were stimulated with either 200 kDa HA (5 mg/ml) or dynorphin A as indicated. (**a**) [Ca^2+^] values, (**b**) percentage of probe discharged during the stimulation. (**c–d**) rat DRG neurons were transfected with the promiscuous G protein Gα_qi5_, loaded with Fura-2/AM and stimulated with either 200 kDa HA (5 mg/ml) or dynorphin A as indicated (**c**) in presence of extracellular Ca^2+^ or (**d**) first stimulated in a medium without [Ca^2+^] (with EGTA 100 µM), and then with the addition of Ca^2+^ to the extracellular medium. The kinetic behavior of the cytosolic Ca^2+^ response is presented as the 340 nm/380 nm ratio.

As shown in Fig. 6, HA is able to induce both mitochondrial (Fig. 6a) and cytosolic (Fig. 6b) Ca^2+^ response in rat DRG neurons expressing the promiscuous G protein Gα_qi5_. The selective action of HA on the KOP receptors was confirmed by the inhibition of Ca^2+^ responses in the presence of norBNI (data not shown).

Finally, we investigated the contribution of intracellular and extracellular Ca^2+^ in rat DRG neurons after HA stimulation. In these experiments (Fig. 6), the cells were first stimulated in a medium without [Ca^2+^] (with EGTA 100 µM), under those conditions the increase of [Ca^2+^] was due only to the release of Ca^2+^ from the intracellular stores. The following addition of Ca^2+^ to the extracellular medium caused a second smaller [Ca^2+^] rise due to the influx through the plasma membrane channels induced by the Ca^2+^-depletion of the intracellular Ca^2+^ stores.

## Discussion

Native HA is a large glycosaminoglycan with repeating disaccharide subunits of N-acetylglucosamine and D-glucuronic acid [32]. It plays a key role in the structure and organization of the ECM and can be found in most organs and tissues [33]. A large abundance of HA is found in the joint synovial fluid [34]. In this context, its unique rheological properties render it highly viscous and lubricating, which are both essential requirements for the integrity of articular cartilage and the overall mechanical performance of synovial joints [35]. In diseases of chronic articular inflammation, the concentration and average MW of HA have been shown to decrease significantly, resulting in reduced synovial fluid (SF) viscosity and possibly cartilage disruption [36]. OA is one of the most common of these synovial joint diseases that is also one of the principal causes of chronic pain in the elderly [37].

VS is an approved treatment modality for OA. Administration of HA preparations to the joint synovial fluid restores the biological properties of normal hyaluronic acid in the ECM, providing pain relief and increasing knee joint mobility [38]. There are several formulations of viscosupplements of varying molecular weights produced by different manufacturers, yet the biological mechanisms responsible for their analgesic activity are still unclear.

The aim of the present study was to determine the activity of HA on OPr on *in vitro* model. We decided to couple the activation of OPr to Ca^2+^ signals using CHO cell lines expressing the promiscuous G protein Gα_qi5_, which can couple any GPCR to an increase in [Ca^2+^]_c_ [17]. The rise in [Ca^2+^]_c_ results in a rapid and transient increase in the [Ca^2+^]_m_ that exceeds the concentration in the cytosol by at least one order of magnitude [26]. The assay technologies that measure the activation of heterotrimeric G proteins by GPCRs are well established within the pharmaceutical industry and are used for the pharmacological study of both natural and surrogate receptor ligands [39].

Aequorin-based assays have been successfully applied for screening of agonists, antagonists and allosteric modulators for different families of GPCRs. This is because aequorin has a number of advantages over other Ca^2+^ indicators: the light emitted does not required any excitation, which eliminates issues related to auto-luminescence (and auto-fluorescence), and background measurements of aequorin are close to zero because mammalian cells are not naturally endowed with chemiluminescent proteins [23].

The wide dynamic range (between 50 nM and 500 µM), together with the low buffering effect, makes the aequorin probe the tool of choice when quantitatively estimating large [Ca^2+^] increases that occur in some cell types. The use of aequorin is now widely accepted as a method to identify agents able to induce GPCR activation [39]. We have used this technique to elucidate important aspects of intracellular Ca^2+^ homeostasis in different pathophysiological contexts [40]–[49]. Thus, we have applied this technology (in combination with the use of the Fura-2/AM Ca^2+^ dye) to identify if OPr are activated by HA.

Experiments performed in CHO cells expressing OPr clearly demonstrated that HA with a medium MW (i.e., 200 kDa) is able to selectively activate the KOP receptor acting as partial agonist. Antagonist experiments performed with norBNI displayed superimposable potency values against HA and dynorphin A, which supports the hypothesis that the effects of HA are due to the selective activation of the KOP protein [13], [50]–[53]. Interestingly, HA was also able to induce a Ca^2+^ response in cells endogenously expressing KOP receptors such as the rat DRG neurons.

Considering that no interesting data were obtained regarding the DOP, MOP and NOP receptors along with the fact that KOP receptors are expressed on fibroblast-like synoviocytes [53], our results corroborate with the observation that HA acts upon KOP and not upon the other OPr. The answer to why HA does not affect the MOP receptor could be due to the different conformational structures of HA compared to morphine, as HA could be have a structure more closely related to dynorphin A.

In conclusion, the present results indicate that HA is able to activate the KOP receptor. The approaches presented in this study are the canonical procedures used for the identification of new ligands for specific receptors as well as in receptor deorphanization programs. Future experiments that involve testing peripheral terminal function such as the inhibition of neuropeptide release from peripheral and central terminals (spinal cord) of primary sensory neurons (nociceptors) will reveal the physiological relevance of this study. Moreover, by using opioid receptor knockout animals, it will be possible confirm that HA selectively affects KOP, as shown in this study through the use of the KOP receptor antagonist norbinaltorphimine.

## Supporting Information

Figure S1 Representative traces of Fig. 4 for some concentrations of agonists and antagonists.
(TIF)
