# Supplementary figures and images for: Hyaluronic Acid Induces Activation of the κ-Opioid Receptor
Source: PLoS One. 2013 Jan 28;8(1):e55510. doi: 10.1371/journal.pone.0055510 (PMC3557250; doi:10.1371/journal.pone.0055510)

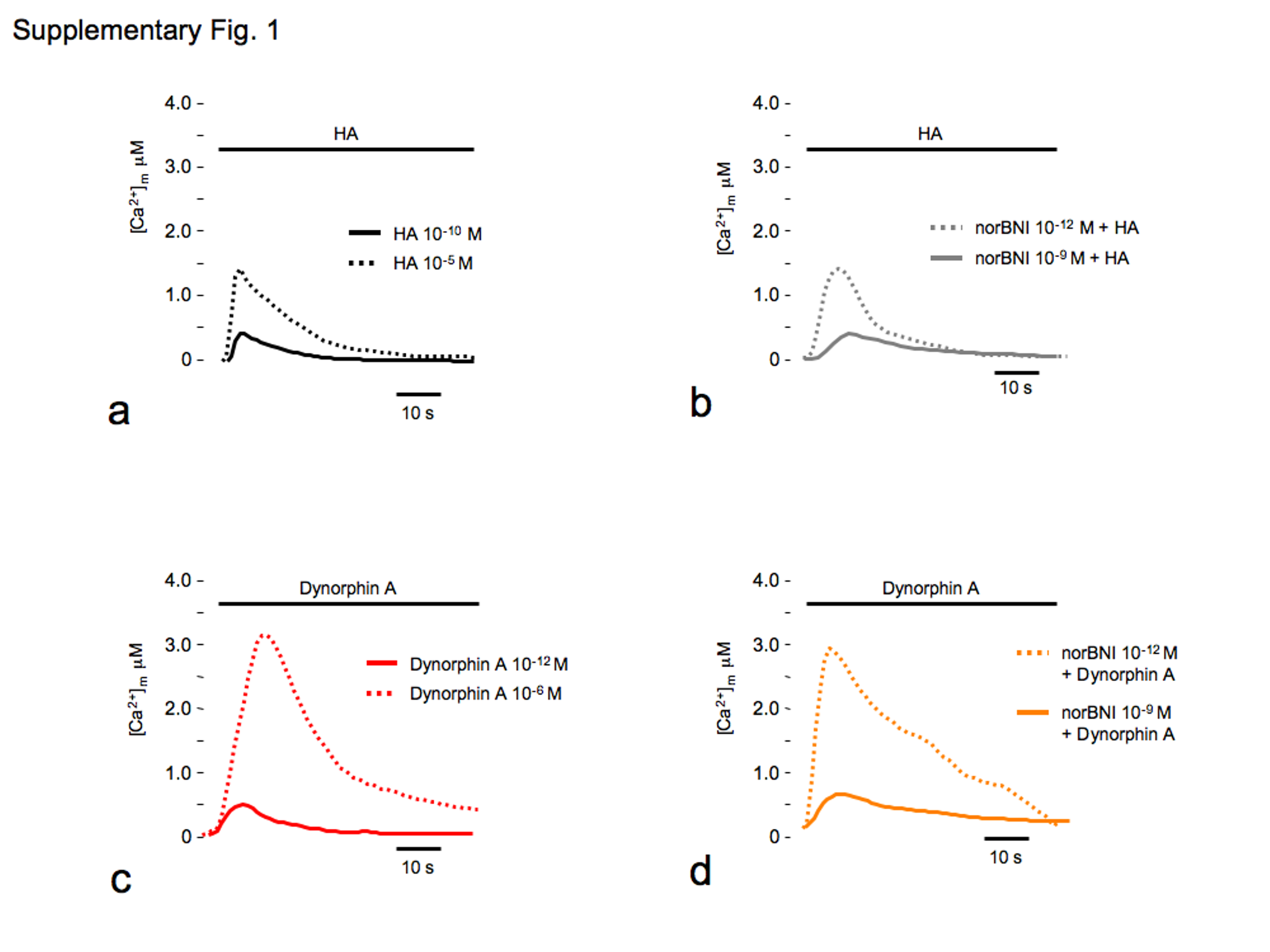

Supplement: Figure S1 — Representative traces of Fig. 4 for some concentrations of agonists and antagonists. (TIF) [file pone.0055510.s001.tif]
